# The significance of molecular heterogeneity in breast cancer batch correction and dataset integration

**DOI:** 10.1186/s13058-025-02159-7

**Published:** 2025-12-24

**Authors:** Nicholas Moir, Dominic A. Pearce, Simon P. Langdon, T. Ian Simpson

**Affiliations:** 1 Applied Bioinformatics of Cancer, University of Edinburgh Cancer Research Centre, Institute of Genetics and Cancer, Edinburgh, UK; 2 Information Services, Research Services, University of Edinburgh, Edinburgh, UK; 3 Fios Genomics, Bioquarter, 13 Little France Rd., Edinburgh, UK; 4 Edinburgh Cancer Research and Edinburgh Pathology, Institute of Genetics and Cancer, University of Edinburgh, Edinburgh, UK; 5 School of Informatics, University of Edinburgh, Edinburgh, UK

**Keywords:** Breast cancer, Gene expression, Dataset integration, Biomarker fidelity, Batch correction, Molecular heterogeneity

## Abstract

**Background:**

Breast cancer research benefits from a substantial collection of gene expression datasets that are commonly integrated for subsequent computational analysis. Gene expression batch effects arising between experimental batches, where technical signal differences confound true biological variation, must be addressed when integrating datasets. Several approaches exist to address these batch effect differences.

**Findings:**

This brief report clearly demonstrates that popular batch correction techniques can significantly distort vital biomarker expression signals. Through the implementation of batch correction and visualisation of integrated expression values, we profile the extent of these distortions and evaluate different variations of Combat batch correction on key breast cancer biomarker expression values.

**Conclusions:**

The diversity of breast cancer as a molecularly heterogenous disease is well recognised. The potential impact of this heterogeneity on vital dataset processing and downstream evaluation remains under-evaluated. We believe this short study presents the first analysis of the interplay between dataset molecular composition and concomitant robustness of integrated, batch-corrected expression signal of breast cancer biomarker expression.

**Supplementary Information:**

The online version contains supplementary material available at 10.1186/s13058-025-02159-7.

## Main text

The reliability and robustness of oncology gene expression profiling studies are affected by size and quality of available sample expression data. Dataset sample count has a major impact on robustness of clinical gene expression analyses, yet the size of most previous studies has been driven by sample availability and processing cost. Nonetheless, enhanced biological insight can be empowered by integrating discrete datasets into a larger meta-dataset. The enhanced statistical power afforded by integrated analysis has been well demonstrated [1, 2] and is increasingly applied in contemporary machine learning and artificial intelligence frameworks [3] to identify and classify molecular events in breast cancer [4] and other malignancies [4–7] that may otherwise be misclassified or remain undetectable in smaller cohorts. Direct integration of probe or transcript-level expression data from multiple studies is therefore potentially very powerful, but technical batch-effects, manifesting both within and between studies, must be addressed before initiating integrated analysis [1, 8–10]. Several groups have investigated optimal batch correction approaches, where expression data are augmented to remove batch effects while ensuring maximal retention of signals representative of true biological variation. To this end, ourselves and others have previously stated that breast cancer datasets should only be integrated where they are suitably ‘similar’ [11], but enhanced understanding of these fundamental issues remain elusive many years later. Additionally, we have cautioned that the composition of breast cancer expression data does not accurately reflect the diverse transcriptomic profiles, or molecular heterogeneity, of breast cancer that drive differences in biology, prognosis, and therapy response at the population level [12], and we have previously highlighted that integrating datasets with vastly varying molecular compositions can dramatically reduce accuracy of prognosis predictions [11].

Batch-effect correction has gained acceptance as a necessary dataset integration step, but there remains little focus on the potential interplay between sample molecular subtype composition and batch correction robustness. In this study, we explore the effect of ComBat [13] batch correction on the fidelity of key breast cancer biomarker expression signals, and show that consideration of molecular heterogeneity is required to ensure optimal preservation of vital gene expression signal integrity.

METABRIC data were collected between 1977 and 2005 from five centres across the UK and Canada and packaged as two stand-alone discovery (*n* = *997*) and validation *(n* = *995)* [14] datasets. With consortium approval, expression data are accessible either as raw Illumina IDAT files or, commonly, and as utilised in this study, log₂-quantile normalised probe-level matrices. While standardising variance within each dataset, the separate pre-normalisation of these expression matrices derives distinct cohort expression medians and quantiles, and is widely accepted as being a major contributor to observed systematic batch effects between datasets. The potential integration of these into a single 1992 sample dataset is of obvious analytical benefit in downstream molecular subtype and novel biomarker discovery pipeline workflows [15, 16], but the existence of strong batch effects between the discovery and validation quantile normalised datasets has been cautioned against in published studies [15, 17]. Samples clearly group according to dataset batch, whereby one could reasonably expect samples to cluster according to PAM50 molecular subtype in the absence of batch effects. The known, strong batch structure in normalised METABRIC data presents an obvious demonstration of how batch effects can dominate true biological signal, but also a real-world opportunity to evaluate and compare batch correction strategies.

Effective batch correction removes technical differences without undesirable further modification of biological signal. Here, we evaluate the performance of several different ComBat implementations on expression values of the ESR1, ERBB2 and AURKA probes. These genes are recognised as key breast cancer biomarkers of hormonal signalling and proliferation [18, 19]. To investigate and illustrate augmentation of these expression values during batch correction, we compare biomarker expression within the canonical PAM50 molecular subtypes (Basal, Luminal A, Luminal B, Her2 + and Normal Breast-like) between METABRIC discovery and validation datasets. Significant expression differences are expected between uncorrected data given the acknowledged presence of batch effects and, recognising sample variation as a sum of technical and biological differences, insight is gained into the relative effectiveness of each ComBat approach by comparing post-correction expression within each molecular subtype class. Because PAM50 subtype assignment captures substantial inter-sample molecular differences, the reduction of variation within subtype classes provides a useful proxy for evaluating batch correction efficacy.

We compare the effect of four different ComBat implementations on biomarker expression. ComBat implements a location-scale (LS) method, which models expression mean and variance within each batch, before adjusting expression according to these models. Default parametric correction assumes the location batch effect variables originate from the same normal distribution and scale effect variables from the same inverse gamma distribution. Non-parametric correction, which uses a Monte Carlo integration-based technique to estimate the location and scale batch effect parameters [20] is also evaluated. In addition to these parametric and non-parametric corrections where the model attributes systematic differences across batches to technical noise, we include two approaches that account for dataset molecular heterogeneity by leveraging a priori PAM50 subtype assignment. We investigate including PAM50 molecular subtype as a covariate in the linear model used by ComBat and additionally introduce a further approach that leverages a pre-correction subtype stratification of samples. While each of these subtype-aware approaches leverages PAM50 subtype, they have crucial differences in implementation and potential signal augmentation. Inclusion of relevant biological covariates, such as molecular subtype, within a design matrix has been suggested as superior at preserving biological signal [20] and has become the preferred and increasingly recommended modern practice when doing batch correction in breast cancer [21].

Finally, we also evaluate subtype-stratified batch correction, which involves pooling samples of each molecular subtype from each batch and correcting each PAM50 molecular subtype group separately. Following this approach, data is split by subtype and independently batch corrected. Each subtype’s corrected data therefore has its own scaling parameters and subsequent cross-subtype comparisons can become unreliable, with potential inter-subtype signal distortion, making this stratified approach suitable only for downstream intra-subtype analysis.

**Fig. 1:**
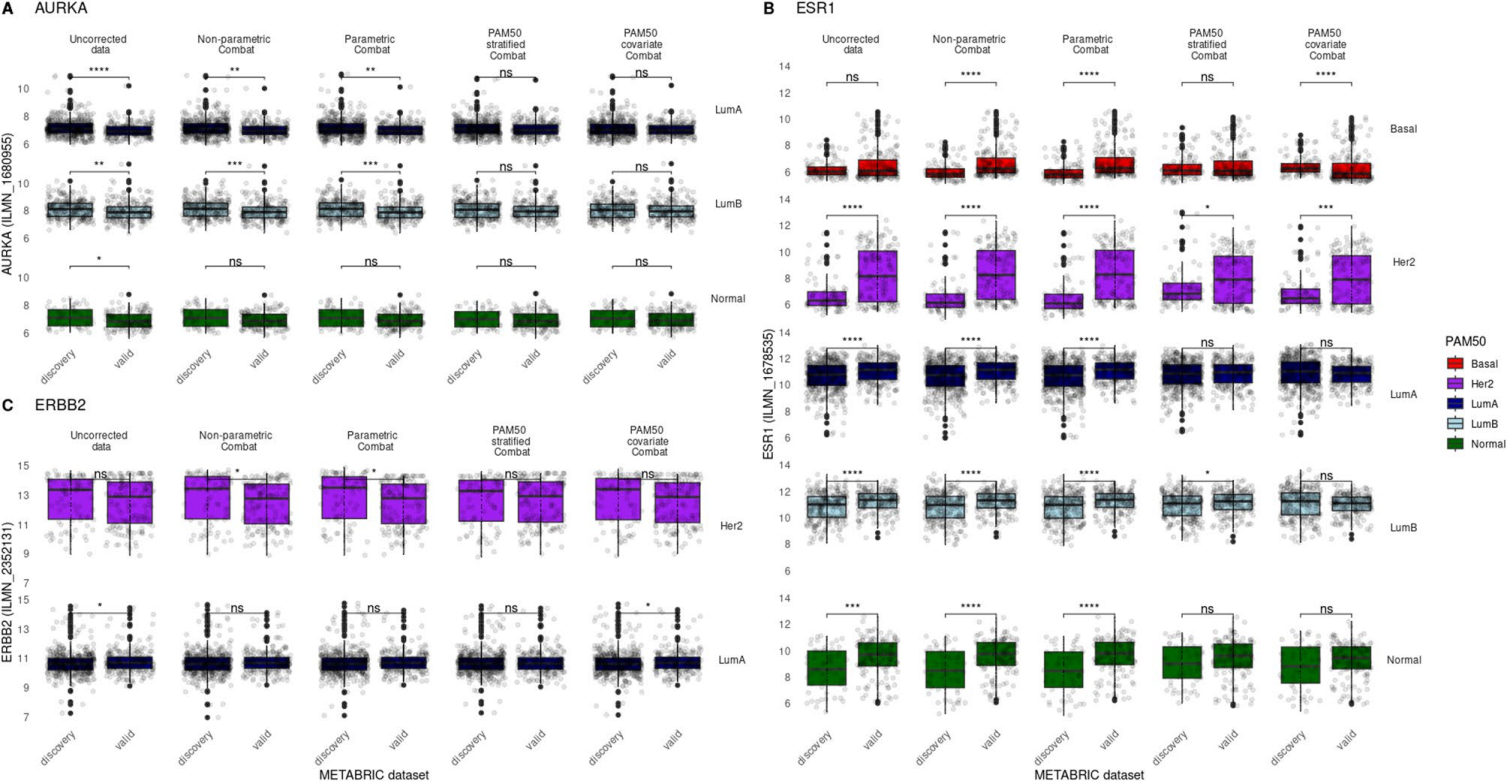
Effect of various ComBat batch correction implementations on METABRIC biomarker expression. Comparison of sample expression values before and after 4 batch corrections in AURKA (**A**), ESR1 (**B**) and ERBB2 (**C**). Expression values are compared between METABRIC discovery and validation batches within PAM50 molecular subtype classifications, allowing visualisation of potential batch correction signal augmentation. Standard non-parametric and parametric correction appear worse at minimising between-batch differences within each PAM50 molecular subtype. The addition of PAM50 molecular subtype as a covariate can improve correction while a stratified PAM50 approach removes batch effects most effectively. Pairwise comparisons between batches were performed with a two-sided Mann–Whitney-Wilcoxon test followed by Holm-Bonferroni correction. Non-significant (p > 0.05) comparisons are unlabelled while * denotes p <  = 0.05, ** denotes p <  = 0.01, *** denotes p <  = 0.001 and **** denotes p <  = 0.0001

Transcriptome biomarker fidelity, represented as a minimisation of within-subtype sample expression difference following correction, clearly appears enhanced when sample molecular heterogeneity is considered during batch correction. Statistically significant (two-sided Mann–Whitney-Wilcoxon test followed by Holm-Bonferroni correction) Aurora Kinase A (AURKA) expression (Fig. 1A) differences persists in both Luminal A (parametric p = 0.001, non-parametric p = 0.001) and Luminal B (parametric p < 0.001, non-parametric p < 0.001) samples following both parametric and non-parametric ComBat correction. In contrast, both a priori PAM50 batch correction approaches remove technical differences and result in no significant population expression differences between discovery and validation sample groups. The benefit of leveraging molecular subtype assignment is further demonstrated with Estrogen receptor alpha (ESR1) expression (Fig. 1B). Notably, uncorrected basal samples showed no significant population differences; however, parametric (p < 0.001), non-parametric (p < 0.001), and PAM50 covariate correction (p < 0.001) each introduced significant expression differences. Only PAM50 stratification preserved the absence of significant differences. While HER2 samples retained strong differences following parametric (p < 0.001), non-parametric (p < 0.001), and PAM50 covariate (p < 0.001) correction, the effect was less pronounced with PAM50 stratified correction (p = 0.019). Correction of ESR1 within the HER2 subtype is further complicated by differences in ER + tumour composition across batches: 27.6% of discovery samples were IHC ER + , compared with 60.5% in the validation set. This indicates that some observed variability reflects true biological differences not fully captured by PAM50 subtypes. In such cases, minimising variability may not be an appropriate objective, and the inclusion of additional covariates could be considered to preserve underlying biology. Within luminal A samples, parametric (p < 0.001) and non-parametric (p < 0.001) perform poorly, while both a priori PAM50 approaches remove significant ESR1 expression differences between sample populations. Similarly, both parametric (p < 0.001) and non-parametric (p < 0.001) fail to resolve population differences in Luminal B samples, with PAM50 stratified correction retaining statistical significance between batches (p = 0.019), and only PAM50 covariate correction removing statistical significance between batches. Parametric (p < 0.001) and non-parametric (p < 0.001) correction additionally performs poorly with normal-like samples, with both PAM50-based approaches removing significant population differences, a prerequisite for accurate post-integration analysis.

The third and final biomarker evaluated is expression of erbB2 receptor tyrosine kinase 2 (ERBB2). Significant expression differences between batches are introduced to HER2 PAM50 samples by parametric (p = 0.011) and non-parametric (p = 0.014) correction. Luminal A samples have between-batch significance removed by all correction techniques except PAM50 covariate correction (p = 0.046).

Leveraging the METABRIC discovery and validation datasets, we have outlined the importance of considering molecular subtype when batch-correcting before dataset integration. An additional interesting scenario arises when an individual dataset is produced at various time-points or by multiple laboratories, introducing potential batch effect manifestation. To evaluate the effect of batch correction in this scenario, we considered a within-dataset evaluation of GSE6532 [22], a published dataset used in multiple studies [23, 24] that has been noted as displaying technical batch effects [24]. Time-dependent batches were identified (see supplemental repository), samples within GSE6532 were ComBat corrected using the four approaches previously described, and biomarker expression values were subsequently evaluated.

**Fig. 2:**
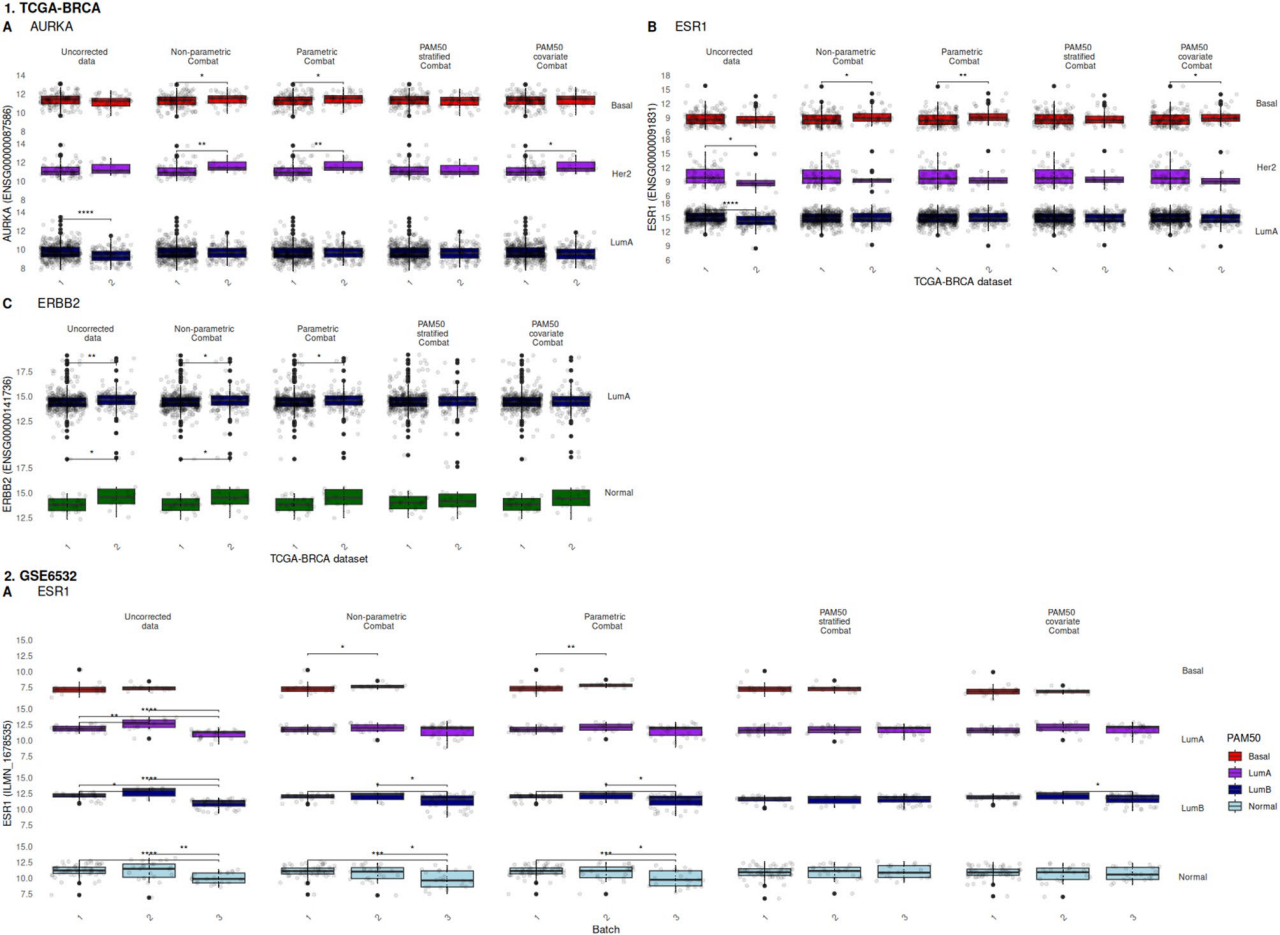
Effect of various ComBat batch correction implementations on 1.TCGA-BRCA and 2. GSE6532 biomarker expression. Comparison of 1.TCGA sample expression in AURKA (**A**), ESR1 (**B**) and ERBB2; and 2. GSE6532 ESR1 (**A**) expression values before and after 4 batch corrections. Expression values are compared between identified batches within PAM50 molecular subtype classifications, allowing visualisation of batch correction signal augmentation. Use of PAM50 sample assignment as a correction covariate or stratifier improves corrected biomarker fidelity. Pairwise comparisons between batches were performed with a two-sided Mann–Whitney-Wilcoxon test followed by Holm-Bonferroni correction. Non-significant (p > 0.05) comparisons are unlabelled while * denotes p <  = 0.05, ** denotes p <  = 0.01, *** denotes p <  = 0.001 and **** denotes p <  = 0.0001

With these expression data, augmentation of GSE6532 ESR1 expression (Fig. 2) displays a similar outcome to METABRIC following batch correction, with fewer significant differences following PAM50 molecular subtype covariate and stratified approaches. Standard parametric and non-parametric ComBat fail to remove technical batch effects with Basal, Luminal B and Normal-like PAM50 molecular subtypes. In this additional dataset, none of the batch-correction methods introduced significant changes in marker gene expression of AURKA and ERBB2 between different batches. The complete biomarker expression analysis for GSE6532 is available in the accompanying online repository. While there is value in batch correction evaluation of this smaller dataset, additional oversight is required when sample size is low. In this instance, ComBat correction can be less stable if a subtype group is small, leading to potential overfitting or undercorrection [20]. Furthermore, the impact of any PAM50 misclassified, or borderline, samples may be amplified and could skew the correction.

In addition to the METABRIC and GSE6532 microarray datasets, we similarly evaluated batch correction of the TCGA-BRCA RNASeq dataset (Fig. 2). In this study, 1180 cancer and adjacent normal breast tissues were collected from 40 tissue source sites and profiled over a span of 5 years from 2010 to 2014. Samples collected in 2010 and 2011 were profiled using one flow cell chemistry^25^, and the remaining samples were profiled using a second flow cell chemistry. We confirmed evidence of batch effects corresponding to these dates, and found further evidence across all 3 biomarkers of fewer significant differences following PAM50 molecular subtype covariate and stratified approaches.

 The data presented herein illustrates often underappreciated subtleties in batch Effect correction. Despite being a vital prerequisite for powerful data integration analysis, undesirable augmentation of molecular signal has the potential to seriously impact integrated analysis. Departures in molecular expression measurements between batches can be reasoned as composites of technical effects alongside genuine biological differences. While the goal of batch correction is to address the former while retaining the latter, and acknowledging that no method will ever perfectly dissect these components, within this brief report we highlight that additional consideration must be given to potential sub-optimal expression differences that persist following correction. The popular ComBat algorithm typically assumes identical distribution of gene expression across batches, but this assumption can be violated by divergent sample transcriptome profiles characteristic of molecularly heterogeneous cancers such as breast. To investigate this effect, we compared both standard parametric and non-parametric ComBat with PAM50-leveraged correction approaches.

Applying parametric or non-parametric ComBat without covariates in breast cancer datasets risks confounding molecular subtype differences with technical batch effects, leading to attenuation of key biological signals and potentially compromised downstream analyses; inclusion of PAM50 and potentially other relevant covariates is therefore required to capture axes of known variation and preserve molecular heterogeneity.

Our results confirm that encapsulation of molecular heterogeneity can help optimise the desired removal of technical effects while preserving true biological signal. Although incorporating molecular subtypes into batch correction pipelines is reasonably expected to mitigate the effects of molecular heterogeneity, its direct impact on breast cancer biomarker expression has, to our knowledge, not been systematically evaluated until now.

In breast cancer transcriptomic studies, the choice of batch correction strategy should be guided by the research objectives and the structure of the data. Inclusion of molecular covariates in the design matrix is generally preferred when the aim is to preserve molecular heterogeneity and enable integrative analyses across subtypes, particularly for biomarker discovery, subtype comparisons, and machine learning applications where statistical stability is critical. By contrast, stratified correction by subtype may be justified in exploratory analyses that focus exclusively on within-subtype biology.

While direct comparison of biomarker expression profiles affords a granular and biologically interpretable assessment of the subtle effects of batch correction, we additionally present PCA visualisations for each analysed dataset to provide a complementary overview of global variance structure (supplementary data).

Robust batch correction in breast cancer requires the incorporation of prior biological knowledge, and this study highlights it as an essential step for preserving true molecular variation and ensuring the validity of genuine molecular signals in heterogeneous breast cancer transcriptomic datasets.

## Supplementary Information

Below is the link to the electronic supplementary material.

Supplementary Material 1.

## Data Availability

METABRIC data are available via committee approval. GSE6532 is freely available at [https://www.ncbi.nlm.nih.gov/geo]. TCGA\_BRCA expression data is freely available. Additional background methodological details are available at [https://github.com/cmpmolonc/BC]. Additional background methodological details are available at https://github.com/cmpmolonc/BC
