# Supplementary material for: The significance of molecular heterogeneity in breast cancer batch correction and dataset integration
Source: Breast Cancer Res. 2025 Dec 24;27:219. doi: 10.1186/s13058-025-02159-7 (PMC12729297; doi:10.1186/s13058-025-02159-7)
Supplement: Supplementary file 1 — Supplementary Material 1. [file 13058_2025_2159_MOESM1_ESM.pdf]

The significance of molecular heterogeneity in breast cancer batch correction and dataset integration

<u>Nicholas Moir, Dominic A. Pearce, Simon P. Langdon, T. Ian Simpson.</u>

<u>Supplementary Material</u>

1. METABRIC PCA plots

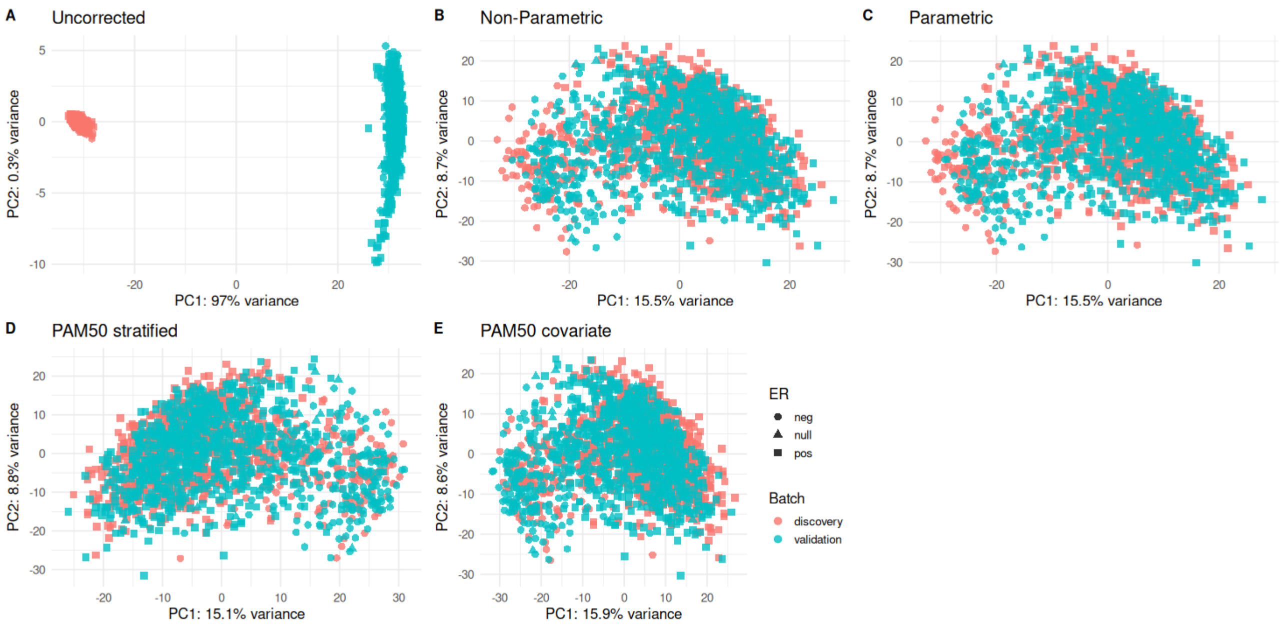

<u>Figure S1. Principal Component Analysis of METABRIC batch correction.</u> The 1000 most variable genes were selected for PCA and the first 2 principal components visualised.

2. GSE6532 PCA plots

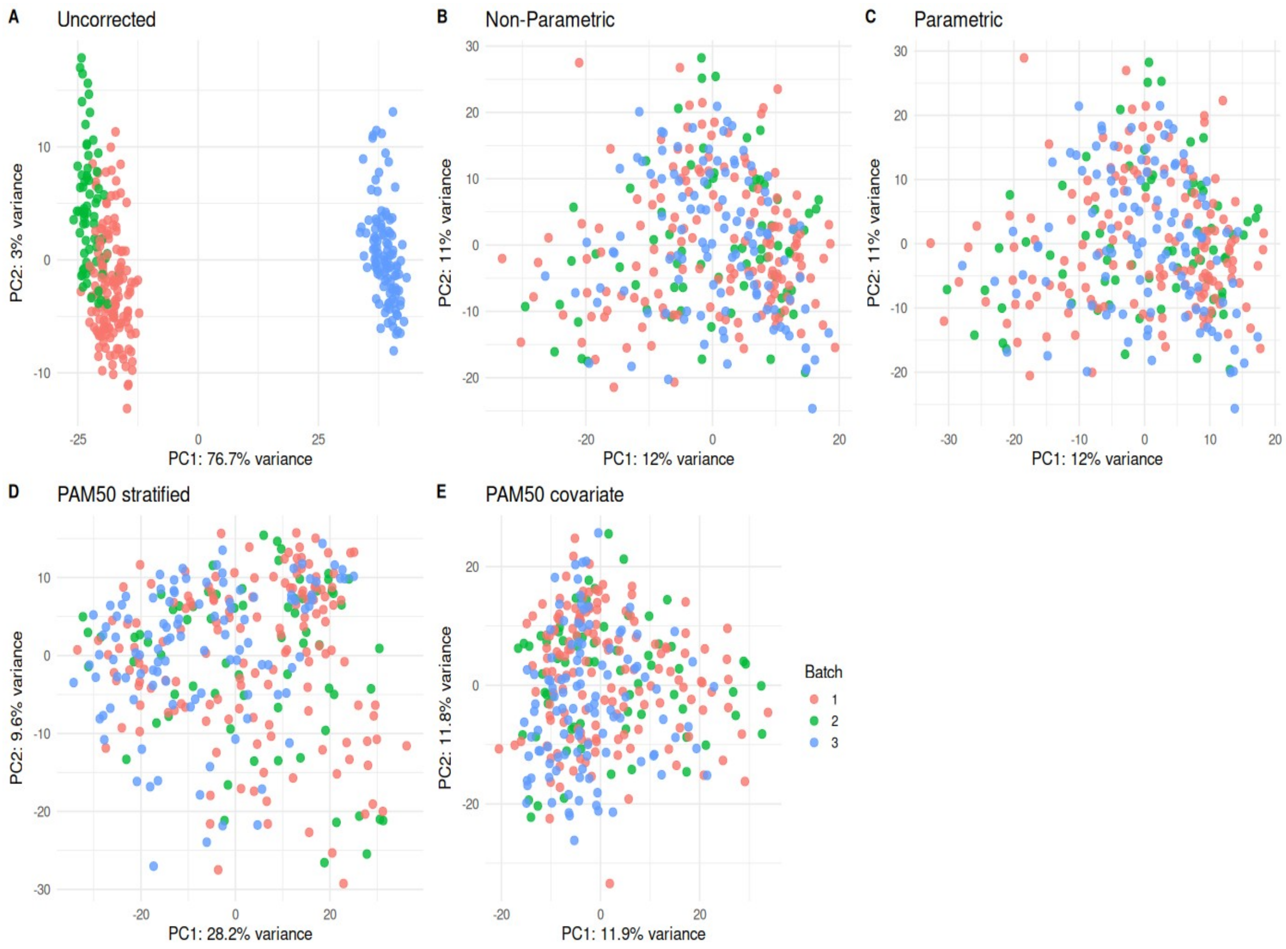

<u>Figure S2. Principal Component Analysis of GSE6532 batch correction.</u> The 1000 most variable genes were selected for PCA and the first 2 principal components visualised.

3. TCGA PCA plots

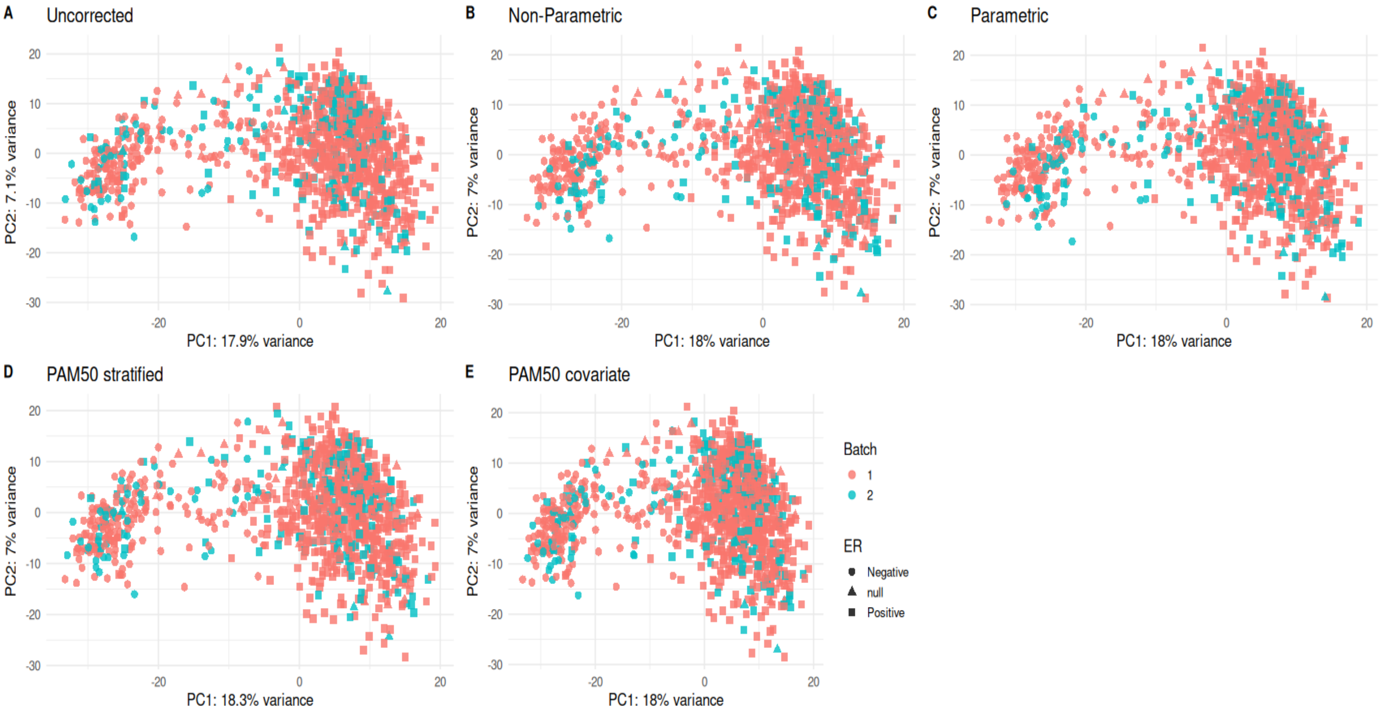

<u>Figure S3. Principal Component Analysis of TCGA-BRCA batch correction.</u> The 1000 most variable genes were selected for PCA and the first 2 principal components visualised.
